# Clinical feature of omicron infection in children with inborn errors of immunity in China

**DOI:** 10.3389/fimmu.2024.1420547

**Published:** 2024-07-23

**Authors:** Han Yang, Fei Sun, Ziwei He, Yan Li, Dan Lu, Tongxin Han, Huawei Mao

**Affiliations:** ^1^ Department of Immunology, Beijing Children’s Hospital, Capital Medical University, National Center for Children’s Health, Beijing, China; ^2^ Ministry of Education Key Laboratory of Major Diseases in Children, Beijing, China; ^3^ Beijing Key Laboratory for Genetics of Birth Defects, Beijing, China

**Keywords:** IEI, COVID-19, children, XLA, SARS-CoV-2, China

## Abstract

**Introduction:**

SARS-CoV-2 infection is hypothesized to be more severe in immunocompromised patients; however, clinical outcomes in children with inborn errors of immunity (IEI) during the Omicron pandemic in China have not been reported.

**Methods:**

This cohort study retrospectively reviewed 71 SARS-CoV-2-infected children with IEI using nationwide data from the National Center for Children’s Health of China. COVID-19 was diagnosed by a positive rapid antigen or nucleic acid test result.

**Results:**

Among 71 SARS-CoV-2-infected children with IEI, male preponderance (male: female ratio of ~1.8:1), a median age of 8 years (IQR 3–11), and a predominance of antibody deficiency (19/71, 26.8%) were detected. Most of the patients got infected through household transmission, while a small proportion of them did so during hospital visits. The mean time periods were 3.3 days (n=44) for incubation, 8.4 days for symptoms (n=69), and 8.8 days for viral shedding (n=37). The time to viral shedding was proportional to the symptomatic period (R^2 =^ 0.1243, p=0.0323) and prolonged in children with X- linked agammaglobulinemia. The most common symptoms of COVID-19 were fever, and some children showed only aggravation of the underlying disease. 15% of IEI children progress to pneumonia, 85% require medication, 17% are admitted to hospital, and 4.1% are classified as critical. Previously application of anti- infective medications was associated with an increased risk of hospitalization after COVID-19 infection. Of the 71 children with IEI, all recovered from COVID- 19.

**Conclusion:**

Overall, Omicron variant did not cause significant life-threatening infections among children with IEI in China, and most of them had a good clinical outcome. Nevertheless, these children exhibit an increased vulnerability to higher hospitalization rates, pneumonia, and severe illness compared to the general pediatric population.

## Introduction

In late 2019, a novel human pathogen with clinical manifestations of pneumonia and respiratory failure emerged and rapidly spread (1, 2). Severe acute respiratory syndrome coronavirus 2 (SARS-CoV-2), a single-stranded RNA virus, is the causative agent of coronavirus disease 2019 (COVID-19) and responsible for severe morbidity and mortality (3, 4). During the COVID-19 pandemic, China maintained a “dynamic zero” policy for almost three years, with a strict mandatory embargo and other restrictive interventions. In December 2022, a fundamental change in the strategy was undergone in China, with restrictive interventions being replaced by more targeted control measures. This was followed by the widespread circulation of Omicron variants of SARS-CoV-2 across the country.

Inborn errors of immunity (IEI), previously termed primary immune deficiencies, are genetic disorders that predispose individuals to infections due to developmental and/or functional abnormalities of the immune system (5–7). As a disease model for understanding immune function, IEI has been informative in uncovering several pathogenic mechanisms of COVID-19 (8, 9). The United Kingdom has reported on the outcomes of 60 patients with IEI in the Journal of Allergy and Clinical Immunology, with an overall infection-fatality rate of 20% (10). A couple of studies conducted worldwide demonstrated that IEI patients are more severely impacted by COVID-19 than immunocompetent individuals, with higher hospitalization rates, younger age at death, longer duration of illness, and extended clearance times for the virus (11–18). However, some studies have reported a mild disease course for COVID-19 in IEI patients (19, 20), with an interesting finding that defects in type I interferon signaling may increase the risk of severe COVID-19 (19, 21–25).

Unquestionably, the robust zero COVID-19 policies in China shielded susceptible populations, such as immunodeficient patients, from five global waves of COVID-19 and avoided large numbers of severe infections with the original strains and delta variants of SARS-CoV-2 (26). However, when these policies were ended, strains BF.7 and BA.5.2 emerged and become the predominant co-circulating strains (27, 28), from which IEI children were not protected. Accordingly, this retrospective study aimed to evaluate the impact of COVID-19 in children with IEI, thus providing the first comprehensive overview of the course, severity, and outcome of COVID-19 among Chinese IEI patients. This study will be a critical reference for clinicians and parents in the management of COVID-19 in children with IEI.

## Methods

### Study design and participants

In early December 2022, the government unveiled 10 prevention and control measures to further optimize its COVID-19 response; however, these new measures resulted in the rapid spread of the SARS-CoV-2 Omicron variant across China. Immunodeficient individuals, such as IEI children, were particularly vulnerable to successive infections. The Department of Immunology at Beijing Children’s Hospital created a case series to summarize information on clinical outcomes following SARS-CoV-2 infection in Chinese IEI patients through online consultation, questionnaires, and telephone follow-ups. The information collected mainly covered personal information, the underlying IEI disease status, SARS-CoV-2 vaccination status, and the clinical features of the infection (e.g., exposure history, symptoms, and treatment). Immunosuppressive agents primarily encompass cyclosporine, tacrolimus, and biologics, among others. Patients aged <18 years with definitive diagnosis of IEI and COVID-19 were eligible for inclusion. COVID-19 was diagnosed by a positive rapid antigen test (RAT) or nucleic acid test (NAT) result. Furthermore, given that pathogen testing was not a mandatory requirement for individuals suspected of infection in China during the period in question, if the patient was in home-based isolation with an exposure to a family member of a confirmed COVID-19, this individual was incorporated into the study in accordance with the World Health Organization’s (WHO) criteria for suspected cases. In China, strain-specific testing is not routinely conducted. During the study period, the predominant circulating variants in China were Omicron, with 90% being the BA.5 and BF.7 sublineages (27). The incubation period refers to the time between when the IEI child was exposed to SARS-CoV-2 and when symptoms first appear. The duration of viral shedding refers to the time from their first SARS-CoV-2-positive NAT/RAT test result to the first negative test result.

### Statistical analysis

Descriptive statistics, non-parametric two-tailed Mann–Whitney U tests, and χ2 or Fisher’s exact tests were applied and p values < 0.05 were considered statistically significant. The Shapiro–Wilk test was used to assess the normality of the data. Age was rounded to the nearest month. All analyses were calculated using GraphPad Prism 9.0 software (https://www.graphpad.com).

## Results

### Characteristics of the study participants

In total, 71 children were enrolled in this study from December 1, 2022, to February 9, 2023. Among these children, 27 types of IEI diseases were identified, with X-linked agammaglobulinemia (XLA) accounting for the largest proportion (10/71, 14%). In terms of the distribution of 10 IEI categories, all the IEI patients except of two children were classified into seven categories, according to the IUIS-2022 classification system (29). Among them, auto-inflammatory diseases (21/71, 30%) were the most frequent category, and defects in intrinsic and innate immunity (4/71, 6%) were the least frequent in this study. Among the included patients, age was non-normally distributed (W=0·9567; p=0.0154) (**Table 1**). The median age of the study population was 8 years (IQR 3–11), ranging from 3 months to 18 years. Sixty-five percent (46 of 71) of individuals were male. Of the 71 participants, 26 (36.6%) had received at least one dose of the inactivated COVID-19 vaccine, and 16 (22.5%) had received two doses. All individuals were deemed to be infected with SARS-CoV-2 and were included in the final analysis. In detail, 70% (50 of 71) of SARS-CoV-2 infections were confirmed by NAT (16 of 71) and/or by RAT (37 of 71). The remainder developed symptoms associated with exposure to COVID-19 patients but were not NAT/RAT confirmed, owing to the non-mandatory status of testing. They were diagnosed to have COVID-19 in accordance with the World Health Organization’s (WHO) criteria for suspected cases.

**Table 1:** Demographic data and the diagnosis of COVID-19-positive patients with IEI.

IEIs of Different Categories		n	Age (y)	Male, n (%)	NAT positive, n (%)	RAT positive, n (%)	Absence of NAT/RAT, n (%)
Total IEIs		71	8 (3–11)	46 (65%)	16 (23%)	37 (52%)	21 (30)
I. Combined immunodeficiencies		7	3 (1–9)	5 (71%)	1 (14%)	1 (14%)	5 (71%)
	Combined immunodeficiencies	5	5 (1.17–10)	4 (80)	1 (20)	0	4 (80)
	Severe combined immunodeficiencies	2	2 (1–3)	1 (50)	0	1 (50)	1 (50)
II. Combined immunodeficiencies with syndromic features		6	5 (3.15–9.5)	5 (83%)	2 (33%)	1 (17%)	3 (50)
	Hyper IgM syndrome	2	8 (5–11)	2 (100)	1 (50)	0	1 (50)
	Hepatic veno-occlusive disease with immunodeficiency	1	9	0	1 (100)	0	0
	Wiskott–Aldrich syndrome	3	4 (0.6–5)	3 (100)	0	1 (33%)	2 (67%)
III. Predominantly antibody deficiencies		19	9 (6–11)	14 (74%)	8 (42%)	14 (74%)	0
	Agammaglobulinemia (AR)	3	7 (3–10)	3 (100)	2 (67%)	2 (67%)	0
	Activated p110δ syndrome (APDS)	1	11	1 (100)	1 (100)	0	0
	Common variable immune deficiency (CVID)	3	11 (1–18)	0	1 (33%)	2 (67%)	0
	SIFD	2	3.67 (0.33–7)	1 (50)	1 (50)	2 (100)	0
	X-linked agammaglobulinemia (XLA)	10	9.5 (7.5–13.3)	9 (90)	3 (30)	8 (80)	0
IV. Diseases of immune dysregulation		6	7.5 (3.75–17)	4 (67%)	0	2 (33%)	4 (67%)
	Autoimmune lymphoproliferative syndrome (ALPS)	3	9 (3–17)	2 (67%)	0	1 (33%)	2 (67%)
	IPEX	1	17	1 (100)	0	0	1 (100)
	LRBA deficiency	1	4	1 (100)	0	0	1 (100)
	STAT3 GOF	1	6	0	0	1 (100)	0
V. Congenital defects of phagocytes		8	5.5 (2–10.5)	6 (75%)	1 (13%)	2 (25%)	5 (63%)
	Chronic granulomatous disease	6	5 (1.75–9.5)	6 (100)	1 (17%)	1 (17%)	4 (67%)
	Severe congenital neutropenia	2	8.5 (3–14)	0	0	1 (50)	1 (50)
VI. Defects in intrinsic and innate immunity		4	7 (3–11)	0%	0	1 (50)	1 (50)
	STAT1 deficiency	1	11	0	0	1 (100)	0
	Tyk2 deficiency	1	3	0	0	0	1 (100)
VII. Autoinflammatory diseases		21	7 (4–12.5)	12 (57%)	3 (14%)	15 (71%)	3 (14%)
	ADA2 deficiency	1	0.75	0	0	1 (100)	0
	Familial cold autoinflammatory syndrome 2	2	5.13 (0.25–10)	2 (100)	0	2 (100)	0
	Blau syndrome	1	4	0	0	1 (100)	0
	Familial Mediterranean fever	1	10	1 (100)	0	1 (100)	0
	A20 haploinsufficiency	1	7	1 (100)	1 (100)	0	0
	Hyper IgD syndrome	1	7	1 (100)	0	0	1 (100)
	PAPA syndrome	2	7.5 (3–12)	1 (50)	1 (50)	1 (50)	0
	PLAID	1	4	0	0	1 (100)	0
	Uncategorized	11	12 (6–14)	6 (55%)	1 (9%)	8 (73%)	2 (18%)
XI. Uncategorized IEIs		2	16 (15–17)	0%	1 (50)	1 (50)	0

NAT, nucleic acid test; RAT, rapid antigen test. SIFD, sideroblastic anemia with immunodeficiency, fevers, and developmental delay; IPEX, immune dysregulation, polyendocrinopathy, enteropathy X-linked; PLAID, PLCγ2 associated antibody deficiency and immune dysregulation.

### Duration of incubation, symptoms, and viral shedding

**Figure 1:**
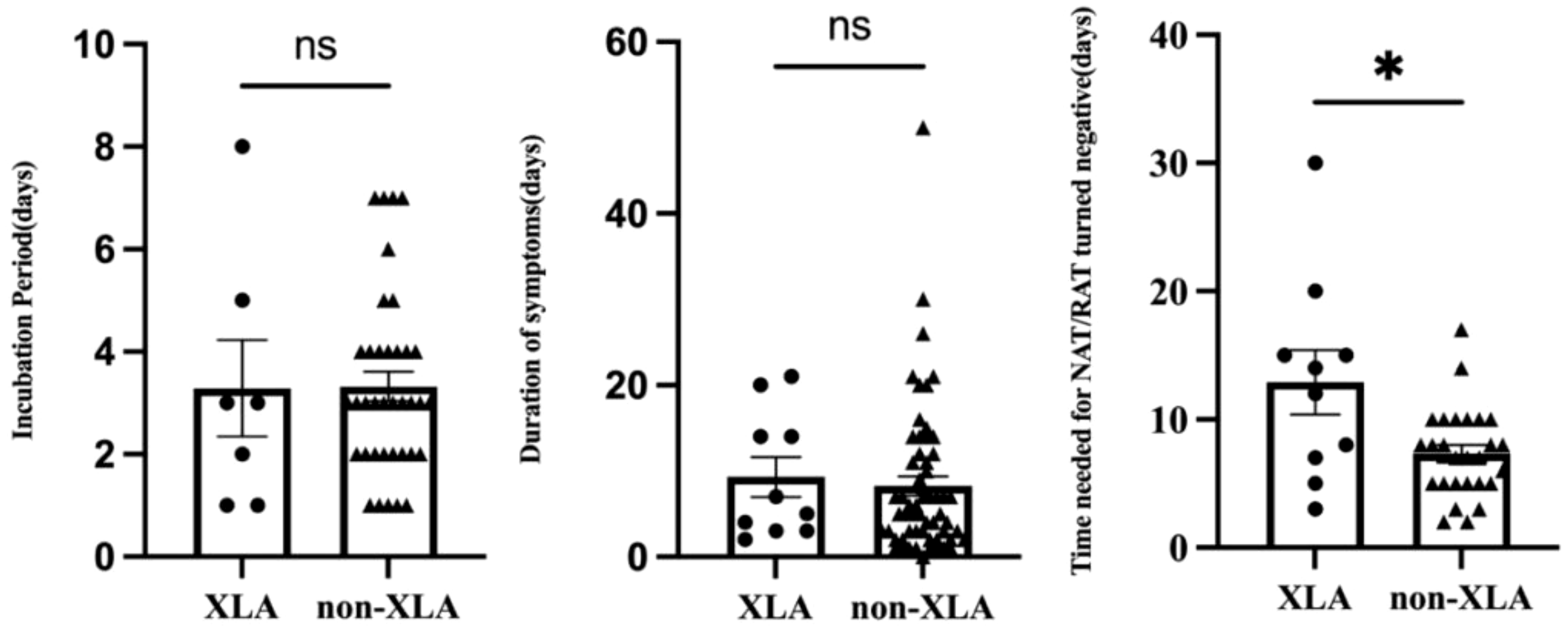
Comparison of the incubation period, duration of symptoms, and time of NAT/RAT conversion to negative between children with XLA and other IEI diseases (non-XLA). In each graph, the dots denote each of the numerical values, the middle long lines denote the mean, and bilateral short lines denote the standard error of the mean. *p=0.0362.

Four children (4 of 71, 5.6%) were infected with the SAR-CoV-2 in the hospital, and the rest (67 of 71, 94.4%) were transmitted through their families. On average, children with IEI showed symptoms 3.3 days after exposure to a positive SARS-CoV-2 patient (n=44) and symptoms lasted 8.4 days before resolving (n=69) (**Table 2**). Not all patients had a clear history of exposure, and two patients had an unclear recollection of the duration of symptoms. First, patients with combined immunodeficiency had a prolonged incubation period compared with other categories of IEIs (4.8 ± 1.6 vs 3.1 ± 1.8, p=0.0405), averaging 1.7 days longer. Second, patients aged 2–5 years experienced a significantly shorter duration (4.7 days) of symptoms compared with the other age groups (4.8 ± 3.3 vs 9.5 ± 9.3, p=0.003). Third, 37 patients monitored the time from their first SARS-CoV-2-positive NAT/RAT test result to the first negative test result, with a mean time to a negative result of 8.8 days, varying from 2 to 30 days. The time of conversion to NAT/RAT-negative was not significantly different among distinct groups of gender, age, SARS-CoV-2 vaccination status, and pre-medication. Furthermore, there was no difference in the duration of viral shedding among different categories of the 10 IEI categories. But in terms of each IEI, it took 5.6 days longer for children with XLA to clear the virus than those with non-XLA IEIs (12.9 ± 8.0 vs 7.3 ± 3.5, p=0.0362). Notably, the significant delay in viral clearance time observed in XLA patients may indicate the pivotal role of antibodies in recovery from COVID-19. No statistically significant differences were found for the virus incubation period or the duration of symptoms between XLA patients and others (**Figure 1**).

**Table 2:** The incubation period, duration of symptoms, and time of NAT/RAT conversion to negative of IEI children following COVID-19.

		Incubation Period		Duration of symptoms		Time needed for NAT/RAT turned negative	
		n	days	n	days	n	days
All		44	3.3 ± 1.9	69	8.4 ± 8.5	37	8.8 ± 5.6
Classification							
	I. Combined immunodeficiencies	5	4.8 ± 1.6*	7	6.1 ± 6.5	2	9.0 ± 1.4
	II. Combined immunodeficiencies with syndromic features	3	2.3 ± 1.2	6	6.3 ± 4.2	3	7.3 ± 2.5
	III. Predominantly antibody deficiencies	15	3.1 ± 2.1	18	8.6 ± 6.8	15	10.7 ± 7.3
	IV. Diseases of immune dysregulation	5	3.6 ± 1.1	6	4.0 ± 2.6	1	5.0 ± 0.0
	V. Congenital defects of phagocytes	6	3.8 ± 2.7	7	8.3 ± 8.6	2	7.0 ± 1.4
	VI. Defects in intrinsic and innate immunity	1	5.0 ± 0.0	2	29.5 ± 29.0	1	10.0 ± 0.0
	VII. Autoinflammatory diseases	8	2.6 ± 0.7	21	8.0 ± 7.8	11	7.8 ± 4.6
	XI. Uncategorized IEIs	1	1.0 ± 0.0	2	18.0 ± 4.2	2	5.5 ± 3.5
Gender							
	Male	31	3.4 ± 1.8	44	8.1 ± 7.5	26	9.4 ± 6.4
	Female	13	3.2 ± 2.2	25	8.9 ± 10.2	11	7.5 ± 2.6
Age (y)							
	<2	6	2.8 ± 1.2	10	7.8 ± 6.3	4	8.0 ± 1.4
	2–5	10	3.8 ± 2.0	16	4.8 ± 3.3*	7	6.7 ± 3.5
	5–10	15	3.6 ± 1.8	20	8.4 ± 7.8	13	7.8 ± 5.0
	>10	13	2.8 ± 2.1	23	11.2 ± 11.4	13	11.2 ± 7.2
Infection sources	Household transmission	42	3.3 ± 1.9	65	8.1 ± 8.5	34	8.7 ± 5.7
	Hospital visits	2	3 ± 0.0	4	13.5 ± 7.6	3	10.7 ± 4.0
SARS-CoV-2 Vaccination							
	No	29	3.6 ± 1.9	43	6.7 ± 5.8	19	7.5 ± 3.7
	Yes	15	2.8 ± 1.7	26	11.2 ± 11.3	18	10.2 ± 6.9
Pre-existing treatment							
	Glucocorticoids	5	2.6 ± 0.5	15	9.7 ± 8.2	9	7.8 ± 3.4
	Immunoglobulin replacement	19	3.1 ± 2.1	21	7.0 ± 6.2	15	9.5 ± 7.4
	Biological agents	3	4.0 ± 2.6	8	7.8 ± 4.8	4	7.3 ± 4.5
	Anti-infective drugs	12	3.9 ± 2.2	16	10.1 ± 7.1	5	8.6 ± 4.4
	Immunosuppressive drugs	12	3.0 ± 1.5	21	10.0 ± 8.6	3	8.2 ± 4.3
	None	4	4.0 ± 0.8	9	11.8 ± 15.7	5	9.4 ± 3.3

*p<0.05.

### Clinical manifestations following SARS-CoV-2 infection

Of the 71 patients, one 2-year-old patient with combined immunodeficiency unexpectedly presented as asymptomatic, while the other patients experienced either mild or severe symptoms. After infection with SARS-CoV-2, the top three most frequently observed symptoms in children with IEI were fever (83.1%), cough (62.0%), and expectoration (33.8%), which was consistent with the general population (**Table 3**). However, the frequency of fever was markedly lower in patients with combined immunodeficiency (category I) than in those belonging to other categories (3/7 vs 59/71, p<0.0001). The other symptoms did not vary across categories of IEIs. In our relatively small sample size, symptoms were similar across the children with various IEI diseases. In addition to the symptoms mentioned in **Table 3**, a 7-year-old patient with sideroblastic anemia with immunodeficiency, fevers, and developmental delay (SIFD) presented with convulsions after 1 day of hyperthermia, despite no history of seizures. He experienced two episodes of seizure within 24 hours: the first lasted 2 minutes and recovered spontaneously; the second lasted ~10 minutes and was resolved with the application of tranquilizer, but tracheal intubation was performed shortly after the onset of acute laryngeal edema.

**Table 3:** The proportions of symptom in each category of IEIs by IUIS-2022 classifications among IEI patients by symptoms.

Classification	I	II	III	IV	V	VI	VII	XI	Total
**n**	7	6	19	6	8	2	21	2	71
**Fever**	42.9%	100.0%	94.7%	83.3%	87.5%	50.0%	81.0%	100.0%	83.1%
**Cough**	71.4%	66.7%	78.9%	50.0%	37.5%	50.0%	52.4%	100.0%	62.0%
**Expectoration**	0.0%	50.0%	52.6%	16.7%	12.5%	50.0%	33.3%	50.0%	33.8%
**Fatigue**	14.3%	33.3%	5.3%	50.0%	0.0%	0.0%	14.3%	50.0%	15.5%
**Stuffy nose**	0.0%	33.3%	21.1%	16.7%	0.0%	0.0%	38.1%	50.0%	22.5%
**Runny nose**	14.3%	33.3%	21.1%	16.7%	0.0%	0.0%	19.0%	50.0%	18.3%
**Sore throat**	14.3%	0.0%	10.5%	33.3%	12.5%	0.0%	19.0%	0.0%	14.1%
**Anosmia**	0.0%	16.7%	0.0%	0.0%	0.0%	0.0%	0.0%	0.0%	1.4%
**Shortness of breath**	0.0%	0.0%	5.3%	16.7%	0.0%	0.0%	0.0%	50.0%	4.2%
**Muscle aches**	14.3%	0.0%	15.8%	33.3%	0.0%	0.0%	9.5%	50.0%	12.7%
**Vomiting**	0.0%	33.3%	5.3%	0.0%	12.5%	0.0%	14.3%	0.0%	9.9%
**Rash**	14.3%	0.0%	0.0%	16.7%	0.0%	50.0%	0.0%	0.0%	4.2%
**Diarrhea**	0.0%	16.7%	0.0%	0.0%	12.5%	0.0%	19.0%	0.0%	8.5%
**Headaches**	0.0%	16.7%	21.1%	33.3%	0.0%	0.0%	9.5%	50.0%	14.1%
**Inappetence**	0.0%	0.0%	5.3%	16.7%	0.0%	0.0%	4.8%	0.0%	4.2%
**Fussiness**	14.3%	16.7%	0.0%	0.0%	0.0%	0.0%	0.0%	0.0%	2.8%
**Joint pain**	0.0%	16.7%	0.0%	0.0%	0.0%	0.0%	4.8%	0.0%	2.8%

The deeper the red, the higher the proportion of IEI patients experiencing the corresponding symptoms.

In addition to the above symptoms associated with SARS-CoV-2 infection, some patients also manifested exacerbation of pre-existing disease. Specifically: 1) a child with common variable immune deficiency (CVID) and another with XLA both presented with aggravation of previous sinusitis; 2) two girls showed recurrence and worsening of a rash: facial herpes in a patient with a STAT3 GOF mutation and thrush in a patient with a STAT1 mutation; 3) a 7-month-old boy with Wiskott–Aldrich syndrome (WAS) showed worsening eczema and markedly reduced platelets, consequently developing rhinorrhea and hematuria; 4) a patient with immune dysregulation, polyendocrinopathy, and enteropathy X-linked (IPEX) due to a FOXP3 mutation showed a sudden increase in liver enzyme levels that had been managed by long-term medication, and a further search for the causative agent revealed a positive nucleic acid test of SARS-CoV-2 infection. Overall, 18.3% of patients showed worsening of their primary disease symptoms following SARS-CoV-2 infection.

Following SARS-CoV-2 infection, 26 individuals underwent chest imaging examination and 11 (11/71, 15%) were diagnosed with coronavirus pneumonia by typical radiographic findings (**Table 4**). Antibody deficiencies (4/11, 36%) predominated in patients who had a diagnosis of pneumonia. The proportion of IEI children older than 10 years with COVID-19 who developed pneumonia was the lowest (1/25, 4%) among all age groups. Overall, however, the incidence of pneumonia did not differ remarkably among distinct groups of IEI classification, gender, vaccination status, and pre-medication.

**Table 4:** The rates of SARS-CoV-2 infection causing pneumonia, requiring drug administration, and requiring hospitalization in children with IEI.

		n	Pneumonic	Hospitalized	ICU admission (n)
All		71	15%	17%	1
Classification					
	I. Combined immunodeficiencies	7	29%	29%	0
	II. Combined immunodeficiencies with syndromic features	6	0%	0%	0
	III. Predominantly antibody deficiencies	19	21%	26%	1
	IV. Diseases of immune dysregulation	6	17%	17%	0
	V. Congenital defects of phagocytes	8	13%	13%	0
	VI. Defects in intrinsic and innate immunity	2	50%	0%	0
	VII. Autoinflammatory diseases	21	10%	10%	0
	XI. Uncategorized IEIs	2	0%	50%	0
Gender					
	Male	46	13%	15%	1
	Female	25	20%	20%	0
Age (y)					
	<2	11	18%	36%	0
	2–5	16	19%	6%	0
	5–10	20	25%	25%	1
	>10	24	4%	8%	0
Infection sources	Household transmission	67	15%	12%	1
	Hospital visits	4	25%	100%	0
SARS-CoV-2 Vaccination					
	No	45	16%	18%	1
	Yes	26	15%	15%	0
Pre-existing treatment					
	Glucocorticoids	15	7%	27%	0
	Immunoglobulin replacement	22	18%	18%	1
	Biological agents	8	0%	13%	0
	Anti-infective drugs	18	22%	33%*	0
	Immunosuppressive drugs	22	18%	18%	1
	None	9	11%	0%	0

*p<0.05.

### Severity, treatment, and prognosis of COVID-19

As a result of COVID-19, 85% (60/71) of children with IEI required medication to control symptoms, 17% (12/71) were hospitalized, and 1% (1/71) were admitted to intensive care (**Table 4**). The patients were all infected in two ways, either at home or in the hospital. No significant differences were found in pneumonia or severe disease rates across transmission modes. Hospital-acquired infections were associated with a higher hospitalization rate (4/4 vs. 8/67, p=0.0005), likely due to the patients having already been hospitalized prior to infection. In terms of treatment, symptomatic drugs (e.g., antipyretics, cough suppressants), antibiotics, and intravenous immunoglobulin were the most commonly used medications, at rates of 81.7%, 18.3%, and 8.5%, respectively. Paxlovid was administered to four children with IEI. A girl with combined immunodeficiency received the drug orally after 3 days of hypothermia and her temperature returned to normal one day later. A 7-year-old boy with a prior diagnosis of SIFD manifested hyperthermia, convulsions, respiratory failure, and severe pneumonia after contracting COVID-19. And the application of antibiotics, gammaglobulin, and invasive respiratory support were ineffective, whereas the initiation of Paxlovid on day 7 post infection resulted in significant improvement of symptoms and chest imaging after five days. The effect of paxlovid in the other two patients with autoinflammatory diseases (AID) was unclear.

The patient population comprises predominantly of antibody deficiency patients. The patients on long-term anti-infective medication before SARS-CoV-2 infection were more susceptible to hospitalization for COVID-19 than those not receiving the medication (p=0.0313). In general, hospitalization rates did not differ greatly by age, sex, diagnosis, classification, or SARS-CoV-2 vaccine status. Respiratory support was utilized in three of the inpatients: one of them was the 7-year-old boy with SIFD mentioned above, who was ventilated with invasive mechanical ventilation and was the only patient admitted to the intensive care unit; the other boy with XLA had a decrease in oxygen saturation to 90% during hospitalization, which returned to normal after the use of nasal continuous positive airway pressure; nasal catheter oxygenation was also applied to a 17-year-old boy. Eventually, all inpatients were discharged from the hospital showing signs of recovery. Follow-up confirmed the absence of clinical symptoms of COVID-19.

## Discussion

To the best of our knowledge, this is the first clinical study in China of children with IEI and COVID-19. As of February 9, 2023, COVID-19 symptoms had resolved in all 71 individuals included in this study, with the only case of death not included in the analysis because of incomplete data. Even though several studies have proposed that immunodeficiency leads to more severe symptoms and a poorer prognosis for COVID-19 (11, 30), the majority of children with IEI in this study did not show severe illness, which would be associated with the varying pathogenicity of the different strains and subtypes of SARS-CoV-2.

In our study, children were most frequently infected with SARS-CoV-2 through family members, which was consistent with previous studies (28, 31–33). A small proportion was infected during regular hospital admission for immunoglobulin or biologic infusions, which, although uncommon, is a unique route of transmission for IEI patients. Therefore, it should be a high priority to protect IEI patients away from household transmission. The provision of stricter isolation measures in hospitals for regularly-admitted patients would be a particularly valuable prevention strategy for patients with IEI but also for those with other chronic diseases. Our findings also demonstrated the low vaccination rate (36.6%) among the IEI participants compared with the general children population aged 3 years and older in China (>90%) and children aged 5 years and older in the USA (85.7%, as of March 8, 2023) (34). Although vaccination status did not correlate significantly with the severity of the current infection, this lack of correlation is likely due to the waning efficacy of the vaccine-induced humoral immune response, given that it had been over six months since the patients received the last vaccination. It has been proven that the COVID-19 vaccine induces immunogenicity in IEI patients (35–39), so increasing the vaccination rate in Chinese children with IEI is imperative.

Our findings indicated that the time taken for viral clearance in children with IEI was prolonged compared with immunocompetent children (8.8 vs 7 days), especially in children with XLA (12.9 days), indicating that antibodies may play a critical role in the process of virus clearance. This was consistent with previous reports of the diminished capacity to clear the virus due to antibody deficiencies (12, 16, 40). The duration of viral clearance in children with IEI, especially XLA, may contribute to increased transmission, highlighting the potential utility of antivirals or other interventions in these patients. Paxlovid can effectively shorten the time to viral elimination in immunocompromised patients (41, 42), and theoretically, early antiviral drug intervention is desirable in immunocompromised patients infected with SARS-CoV-2. Linear regression analysis revealed a positive correlation between the time of NAT/RAT conversion to negative and the duration of symptoms in children with IEI (R2 = 0.1243, p=0.0323). A previous report proposed that the duration of viral shedding is extended in symptomatic patients compared with asymptomatic patients (43), which is comparable with our finding.

Furthermore, the predominant symptoms in IEI children were fever and cough. However, compared with the general population (44), the percentage of children with IEI in our study that presented with fever was lower. Therefore, we need to keep alert of the possibility of asymptomatic infection in such population. In addition, patients with recurrent pneumonia may be overlooked clinically, as the original symptoms and lung pathology obscure the development of COVID-19. On the other hand, in some cases, COVID-19 only aggravates the underlying disease. For example, an IPEX patient only presented with abnormally elevated serum liver enzymes, and a patient with STAT1 mutation manifested with exacerbation and recurrence of thrush. Taken together, suspicion of COVID-19 should be born in mind when examining IEI patients with no apparent infection phenotype. Therefore, in clinical practice, it is easy to overlook asymptomatic carriers and patients who only exhibit exacerbation of their underlying conditions. These individuals may have reduced shedding capabilities, which could increase the risk of disease transmission.

The prevalence of COVID-19 pneumonia (15% vs 48%, p=0.0005) and hospitalization rates (17% vs 63%, p<0.0001) of IEI patients in this study are lower than those in other countries reported before (13, 45). On the one hand, this may be related to the distribution of different disease categories. In other studies, antibody deficiencies (53/94, 56%) were predominant (13), whereas in this study, auto-inflammatory diseases (21/71, 30%) were predominant, implying that the patients in our study may not be as deficient in immune function against the virus. On the other hand, differences in the circulating viral strains may contribute to the differences in clinical manifestations. The pandemic of the Omicron variant in China has generally resulted in mild disease symptoms among the general population. The Chinese National Health Commission cites an 8% rate of pneumonia after infection in the overall general population. However, it is lower than that of pneumonia among children with IEI in this study (11/71, 15%). Furthermore, the incidence of hospitalization and severe illness among patients with IEI surpasses that observed in the general pediatric population during the same period when the particular strain was prevalent in China (46, 47). Taken together, although the Omicron variants have not been conclusively linked to the development of critical infections in Chinese children with IEI, they did show an increased susceptibility to hospitalization, pneumonia, and severe illness when compared with the general pediatric population. Upon completion of the study, it came to our attention that a 17-year-old female with Common Variable Immunodeficiency (CVID) succumbed to severe pneumonia following a COVID-19 infection. Due to the unavailability of comprehensive information, this case was not included in the study.

Our findings indicate that pre-existing antibiotics for other pathogenic infections or prophylaxis in children with IEI are related to a greater tendency to be hospitalized after SARS-CoV-2 infection. It is reasonable to infer that the presence of a prior infection can be a risk factor for severe COVID-19. Regarding treatment in IEI patients, symptomatic drugs such as antifebrile and cough suppressants improved symptoms in a large proportion of patients. The patients given with Paxlovid showed a good response. Despite the small sample size, our finding was consistent with a previous report confirming the effectiveness of Paxlovid in the treatment of COVID-19 in immunosuppressed patients (48). In China, Paxlovid can only be administered to those over 12 years of age, so there is an urgent need to investigate the efficacy of Paxlovid in younger children, in order to apply the new treatment regimens to slow the progression of COVID-19.

Our study provides insights into COVID-19 in Chinese children with IEI. The patients did not develop life-threatening illness, and had full remission. However, the small sample size limits broader applicability. The variability in IEI manifestations across categories underscores the need for personalized treatment strategies. Larger, multicenter studies are essential for robust data to refine these approaches. Advanced immunoassays and genomics could elucidate SARS-CoV-2 pathogenesis in IEI, aiding in targeted therapy. We propose proactive monitoring and treatment to avert severe outcomes, emphasizing vaccination to counteract prolonged viral shedding, a critical factor given antibodies’ role in clearance. This underscores the need for tailored public health measures and therapeutics for IEI patients amidst the pandemic.

This study had a few limitations. First, the validity of some of the data from the questionnaires was dependent on the participant’s responses. Thus, questionable responses were verified to the best of our ability to ensure authenticity. Second, it is conceivable that the actual proportion of asymptomatic patients is higher than in this study since symptomatic patients are more willing to contact a healthcare provider, and testing for COVID-19 is no longer compulsory in China.

In summary, this is the first description of COVID-19 in children with IEI in China. During the Omicron pandemic, most of the IEI patients with SARS-CoV-2 infection did not develop severe illness, and all patients achieved clinical remission. It is crucial to protect children with IEI from infection through household transmission, as it is the main route of infection for IEI patients. The clinicians need to be alert in recognizing COVID-19 infection in children with IEI, as some patients may be asymptomatic or show only worsening of the primary disease after infection. It was found that the longer the duration of symptoms, the longer the time to convert to a negative NAT/RAT result. The prolonged time to clear the SARS-CoV-2 in patients with XLA needs to be emphasized. Finally, the end of the zero COVID-19 policy does not represent the end of COVID-19 in China, and IEI patients remain vulnerable to infection. Although all participants in our study ultimately recovered, the incidence of pneumonia and hospitalization rate remained significantly higher than those in the general population. Moreover, these individuals exhibited a particularly pronounced risk of progressing to severe cases. A further large-scale study of the pediatric population is warranted to obtain the full picture of COVID-19 in children with IEI.

## Data availability statement

The raw data supporting the conclusions of this article will be made available by the authors, without undue reservation.

## Ethics statement

The studies involving humans were approved by Bejing Children’s Hospital Research Ethics Committee. The studies were conducted in accordance with the local legislation and institutional requirements. Written informed consent for participation was not required from the participants or the participants’ legal guardians/next of kin in accordance with the national legislation and institutional requirements.

## Author contributions

HY: Data curation, Investigation, Methodology, Software, Writing – original draft. FS: Data curation, Methodology, Writing – original draft. ZH: Data curation, Formal analysis, Writing – original draft. YL: Project administration, Resources, Writing – review & editing. DL: Writing – review & editing. TH: Methodology, Writing – review & editing. HM: Writing – original draft, Writing – review & editing.
